# Peripheral blood gene expression stratifies rate of progression to type 1 diabetes in autoantibody-positive children in the TEDDY study

**DOI:** 10.3389/fimmu.2025.1703839

**Published:** 2025-11-11

**Authors:** Jia Yi Hee, Yann Abraham, Ahmed M. Mehdi, Kim-Anh Lê Cao, Ranjeny Thomas

**Affiliations:** 1 The University of Queensland Frazer Institute, Faculty of Health, Medicine and Behavioural Science, The University of Queensland, Translational Research Institute, Brisbane, QLD, Australia; 2 Deeplife, Vernon, France; 3 Data Science Collaborative Research Platform, The University of Queensland, Brisbane, QLD, Australia; 4 Queensland Digital Health Centre, The University of Queensland, Brisbane, QLD, Australia; 5 Melbourne Integrative Genomics and School of Mathematics and Statistics, The University of Melbourne, Melbourne, VIC, Australia

**Keywords:** autoimmune disease, gene expression, pathway analysis, protein-protein interaction analysis, seroconversion, type 1 diabetes, type 1 diabetes progression

## Abstract

**Introduction:**

In type 1 diabetes, autoimmune destruction of pancreatic β cells results in insulin deficiency, leading to hyperglycemia. Islet autoantibodies, which precede autoimmune progression, usually develop years before diabetes onset, although some individuals develop diabetes without them. However, not all children who develop islet antibodies progress to diabetes, and they do not all progress at the same rate. Genomic markers may help identify high-risk children for early intervention.

**Methods:**

Using gene expression profiles derived from peripheral blood mononuclear cells collected from 62 high-risk, islet autoantibody-positive children in the TEDDY cohort study, of whom 56 progressed to diabetes, we identified differentially expressed genes, pathways, and protein–protein interactions associated with progression from islet autoantibody seropositivity to the clinical onset of diabetes.

**Results:**

After seroconversion, progressors were distinguished by a peripheral blood gene expression profile enriched for MHC class II-related functions and immune response pathways. Within protein– protein interaction (PPI) networks, we identified SMARCA4 as the central hub and found that its expression stratified progression to type 1 diabetes after seroconversion. Differentially expressed genes in the PPI networks were also highly connected to type 1 diabetes drug–gene targets, particularly JAK2.

**Discussion:**

The enrichment of MHC class II-related functions and immune response pathways after seroconversion highlights immune activation in progressors, whose rate of progression could be stratified as fast or slow based on a 20-gene signature, which warrants confirmation in an independent cohort.

## Introduction

Type 1 diabetes is an endocrine disease involving autoimmune destruction of pancreatic β cells, resulting in insulin deficiency and subsequent hyperglycemia (1). Although type 1 diabetes can be managed, lifelong insulin replacement therapy is required. Consequently, and with the increasing incidence of type 1 diabetes, there is a growing emphasis on investigating primary and secondary preventive interventions for at-risk children with high-risk genetic variants, such as HLA class II and *PTPN22* (2, 3).

Primary prevention strategies target at-risk children before the onset of disease, while secondary prevention strategies target at-risk individuals in whom a subclinical autoimmune process has begun, indicated by the presence of single or multiple islet autoantibodies (stage 1 and 2 diabetes) with or without dysglycemia, but who have not yet presented with diabetes (stage 3 diabetes). Most at-risk children who develop autoantibodies eventually progress to diabetes, albeit at different rates; higher numbers of autoantibodies are associated with faster progression, and as serum autoantibodies generally emerge years before clinical onset, this provides a large window of opportunity for intervention (3–5).

With the advent of immunotherapy that can delay or prevent the onset of type 1 diabetes in children with stage 2 diabetes, islet antibody screening programs are identifying autoantibody-positive (ABP) children. The analysis of peripheral blood RNA transcript levels in at-risk children may highlight key differentially expressed (DE) genes or pathways (6) that can predict progression to type 1 diabetes (7). This predictive power is enhanced when DE genes are analyzed at multiple longitudinal time points, such as from birth until diabetes onset, as exemplified by studies such as The Environmental Determinants of Diabetes in the Young (TEDDY) study, which longitudinally profiled gene expression from peripheral blood mononuclear cells (PBMCs) in children with high-risk genetic variants across Europe and the United States.

In this study, we used longitudinal gene expression data from the TEDDY cohort study to investigate genes and pathways involved in progression from islet autoantibody seropositivity to clinical onset of diabetes in children.

## Materials and methods

### Study design and participant characteristics

The TEDDY study is a prospective study that seeks to elucidate triggers of type 1 diabetes in children. The TEDDY study recruited newborns younger than 4 months of age at first follow-up who were HLA-eligible at genetic screening from six clinical centers located in Denver (Colorado), Augusta (Georgia)/Gainesville (Florida), and Seattle (Washington) in the United States, and in Finland (Turku), Sweden (Malmö), and Germany (Munich) between 2004 and 2009. As participants were recruited from both the general population and first-degree relatives with type 1 diabetes, the study sample is representative of the larger population of interest. TEDDY visits were scheduled every 3 months until 4 years of age. At 4 years of age, children deemed persistently ABP remained on the 3-monthly visit schedule; subsequent visits for all other children occurred every 6 months until 15 years of age or until the occurrence of type 1 diabetes.

PBMC microarray data of participants were mapped to their clinical metadata from both matched islet autoantibody and type 1 diabetes case–control datasets to obtain information on seroconversion and the clinical onset of type 1 diabetes, respectively. Additional data on the age at specific autoantibody onset and family history of type 1 diabetes were requested, enabling the identification of the first-appearing autoantibody and the number of autoantibodies present. Although some participants were followed beyond their initial type 1 diabetes diagnosis, only observations from the time of first autoantibody detection until the onset of diabetes for each participant were included in the analyses. By 6 years of age, participants had either progressed to diabetes or were lost to follow-up. Participants developing autoantibodies that subsequently became seronegative (sero-revertors), likely due to placental transfer of maternal autoantibodies, were excluded. The resulting TEDDY microarray dataset comprised 273 samples collected from 62 seropositive children, of whom 56 progressed to type 1 diabetes.

The microarray experiments identified a total of 47,167 genes that were used for analysis. Details on PBMC preparation and microarray hybridization have been previously described (8). PBMC data from the TEDDY study were accessed with approval from the NIDDK Central Repository GWAS Data Access Committee (phs001442). The study was approved by the Metro South and University of Queensland Human Research Ethics Committees.

### Linear mixed-effect model with spline

The expression of each gene over time in the TEDDY cohort was modeled using a linear mixed-effect model with spline with the *lmms* package (version 1.3.3) in R (9). Linear mixed modeling with spline provides higher sensitivity through the use of spline functions, which offer the flexibility and ability to capture complex or non-linear relationships necessary to handle our data (10).

For this model, the time of sampling for each microarray sample was based on age at sample collection. However, for plotting and visualization purposes in the heatmaps, time was shown relative to the clinical onset of type 1 diabetes. Hence, for children who progressed to diabetes, the time of sampling was calculated by subtracting the age at diabetes diagnosis from the age at sampling. For children who did not progress to diabetes, the time of sampling was calculated by subtracting the mean age at diagnosis of those who progressed to diabetes from the age at sampling. This approach provided standardized time points, making it easier to compare data across participants and over time, as all measurements were anchored to the clinical onset of diabetes.

Gene expression was fitted using a linear mixed-effect model with spline. In the model, the dependent variable was the gene expression value; the fixed-effect variables were time (sampling age), outcome (a two-level factor variable: “progressor” or “non-progressor”), and the time/outcome interaction variable; and the random-effect variable was the individual (a factor variable indicated by the unique identifier of each subject). As there were no significant differences between the two groups for the remaining variables, we did not adjust for them in the model.

We considered the group and group × time effects for each gene, and the associated *p*-values were corrected using the Benjamini–Hochberg procedure. A final significance threshold of a false discovery rate (FDR) < 0.05 was applied in all analyses. *p*-values less than 10^-^³ were reported as *p* < 0.001.

### Weighted gene correlation network analysis

To explore the relationship between genes based on their expression patterns and to identify modules of highly correlated genes, weighted gene correlation network analysis was performed using the **WGCNA** package in R (11). Briefly, pairwise correlations between DE genes were calculated using Pearson correlation to construct a gene co-expression similarity matrix. This matrix was then transformed into an adjacency matrix using a soft-thresholding power (softPower = 3) to emphasize strong correlations while reducing noise. The resulting weighted network was used in weighted gene correlation network analysis to detect modules of highly co-expressed genes. Module eigengenes were correlated with external phenotypic traits to identify biologically relevant modules.

### Functional pathways, protein-protein interaction networks, and cellular deconvolution

Gene set enrichment analysis of DE genes was performed using the Gene Ontology Resource (https://geneontology.org) for biological processes. Kyoto Encyclopedia of Genes and Genomes (KEGG) analysis was performed using the *clusterProfiler* package (version 4.12.6) in R. Significance was determined using adjusted *p* < 0.05. Lollipop plots for gene ontology terms by module were constructed using the *ggplot2* package in R.

Protein–protein interaction (PPI) networks were constructed to elucidate interactions within each module using Cytoscape (version 3.10.3). Full STRING networks were generated with a confidence score cutoff of 0.90, indicating high reliability of the interaction between protein pairs. A maximum of 10 additional interactors was allowed, meaning that up to 10 high-confidence proteins not included in the original input list could be added to the network. These interactors helped complete the network structure and supported the identification of hub genes. The size of each node indicates the degree of the corresponding protein within the network, with larger nodes representing hub proteins that interact with more proteins.

Digital cytometry used a cell enrichment approach, comparing PBMC transcriptional profiles with cell type–specific signatures using the xCell package (version 1.1.0) in R (12). A total of 64 immune and stromal cell types can be inferred using xCell, of which 20 immune cell types of interest were retained. Subsequently, a linear mixed-effects model was fitted to identify DE cell types between progressors and non-progressors.

### Machine learning methods to predict progression rate in progressors

To identify DE genes associated with fast or slow progression to type 1 diabetes, linear mixed modeling with spline was used. Participants were categorized as fast or slow progressors based on the duration between seroconversion and clinical diagnosis of type 1 diabetes. Those who progressed within ≤ 2 years were classified as fast progressors, while those who progressed > 2 years after seroconversion were classified as slow progressors.

To identify gene signatures predicting the rate of progression in progressors, three machine learning models were applied to the top 500 of 1,967 DE genes using gene expression data from blood samples collected closest to, but after, the age of seroconversion. To control for overfitting, model performance was evaluated using leave-one-out cross-validation with the area under the receiver operating characteristic (ROC) curve (AUC) as the performance metric. The models included a Random Forest (500 trees, probability mode), XGBoost (objective = “binary:logistic”, max_depth = 4, η = 0.1, subsample = 0.8, colsample_bytree = 0.8, nrounds = 100), and GLMNET (α = 0, binomial family, λ = λ_min). Feature importance was derived from permutation importance (Random Forest), gain scores (XGBoost), and absolute coefficient magnitude (GLMNET) to identify genes contributing most strongly to progression classification.

R version 4.4.0 was used for all R analyses.

## Results

### Characteristics of seropositive participants and data collected

From the nested case–control TEDDY cohort consisting of 401 participants, we constructed a smaller cohort of 62 ABP participants (**Table 1**), with sero-revertors excluded from analyses (see **Supplementary Figure 1A** for inclusion criteria). Prospective observations for all participants, demonstrating the age at seroconversion and type 1 diabetes onset, are presented in **Supplementary Figure 1B**.

**Table 1:** Characteristics of TEDDY subjects. Age measured in years.

	Total (N = 62)	Progressors (N = 56)	Non-progressors (N = 6)	P-values
No. of microarray datapoints, n	273	255	18	
Sex, n (%)				0.2015
Male	29 (46.8)	28 (50.0)	1 (16.7)	
Female	33 (53.2)	28 (50.0)	5 (83.3)	
Maternal type 1 diabetes, n (%)	8 (12.9)	6 (10.7)	2 (33.3)	0.2531
Paternal type 1 diabetes, n (%)	17 (27.4)	16 (28.6)	1 (16.7)	1
Sibling type 1 diabetes, n (%)	7 (11.3)	6 (10.7)	1 (16.7)	0.5284
No FDR, n (%)	34 (54.8)	31 (55.4)	3 (50.0)	1
Age at type 1 diabetes, median (IQR)	-	2.77 (1.78-3.78)	–	
Age at seroconversion, median (IQR)	1.07 (0.77-1.79)	1.02 (0.76-1.56)	2.03 (1.14-2.60)	0.1189
Multiple autoantibodies, n (%)	57 (91.9)	54 (96.4)	3 (50.0)	0.0078

FDR, First-degree relatives, IQR, Interquartile range. Two-sided statistical significance was assessed using the Chi-square test for categorical data and the Wilcoxon rank-sum test for continuous data.

Among seropositive participants, the sex distribution was approximately equal, with 46.8% males and 53.2% females. A greater proportion had paternal type 1 diabetes (27.4%) compared with maternal type 1 diabetes (12.9%), while 11.3% had an affected sibling. The majority (54.8%) had no first-degree relative with type 1 diabetes. The median (interquartile range, IQR) age of seroconversion was 1.07 (0.77–1.79) years, and the age of diabetes diagnosis was 2.77 (1.78–3.67) years. In total, 91.9% (n = 57) developed multiple autoantibodies, and 90.3% (n = 56) progressed to type 1 diabetes.

### Linear mixed effect modelling with spline identifies differentially expressed genes in PBMC data

Linear mixed-effect spline modeling was used to compare the expression of each gene over time in TEDDY ABP progressors and non-progressors. Genes with group effects indicated significant differences in expression levels between progressors and non-progressors, while genes with group × time effects indicated significant differences in the trajectory of expression levels over time between the two groups. Of the 47,167 genes analyzed, 320 and 26 genes were significantly DE for group and group × time effects, respectively, after false discovery rate (FDR) correction.

**Figure 1:**
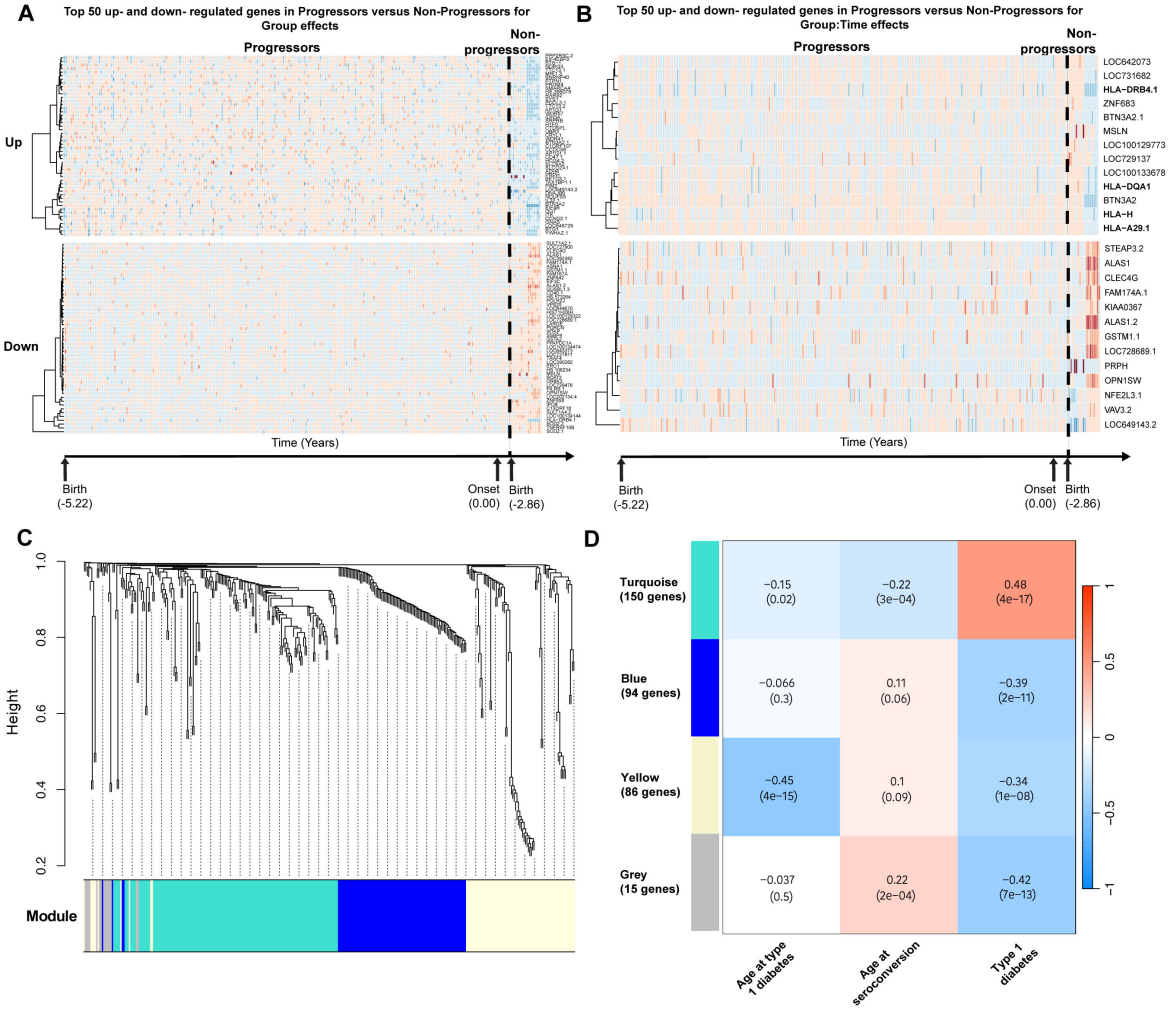
Differential gene expression analyses in TEDDY. Schematic diagram of the cohort and analytical workflow used to obtain DE genes. **(A)** Top 50 most significantly upregulated and downregulated genes significant for group effects. **(B)** Upregulated and downregulated genes significant for group × time effects in progressors versus non-progressors. **(C)** Gene dendrogram generated using average-linkage hierarchical clustering. The colored row beneath the dendrogram indicates module assignments identified by the Dynamic Tree Cut algorithm in WGCNA. Four modules—yellow, turquoise, blue, and gray—were constructed from differentially expressed genes. **(D)** Correlation heatmap demonstrating the relationship between modules and traits of interest. Each cell displays the Pearson correlation coefficient between a module eigengene and a trait, with the corresponding p-value in parentheses.

Using the estimates returned by the model for each gene, DE genes were classified as upregulated or downregulated. Among genes significant for group effects, 112 were upregulated and 208 were downregulated. Among genes significant for group × time effects, 13 were upregulated and 13 were downregulated. In particular, HLA class I and II genes were upregulated in progressors compared with non-progressors. The top 50 genes in each category are presented in the heatmaps in **Figures 1A, B**.

Due to the imbalance in the numbers of non-progressors and progressors, a sensitivity analysis was performed by repeatedly down-sampling (100 times) progressors to match the number of non-progressors while retaining all repeated observations for each selected subject. Of the 320 genes identified as significant for group effects and the 26 genes identified as significant for group × time effects, 80.9% and 69.2%, respectively, remained significant (adjusted p < 0.05) in at least 50% of the 100 resampling iterations.

### Gene modules are correlated with type 1 diabetes phenotypic traits

To identify groups of genes with coordinated expression across samples, weighted gene co-expression network analysis (WGCNA) was applied, resulting in four modules of highly co-expressed genes (**Figure 1C**). The first module (yellow) consisted of 86 DE genes and was negatively correlated with progression to diabetes and age at onset of type 1 diabetes (correlation = −0.45, p < 0.001). The yellow module could be further subdivided, with one subcluster showing a stronger correlation with age at onset of type 1 diabetes and another with disease development (**Supplementary Figure 2**). However, for downstream analyses, the yellow module was retained as a single module.

The second module (turquoise) consisted of 150 genes and was positively correlated with progression to type 1 diabetes (correlation = 0.48, p < 0.001). The third module (blue) consisted of 94 genes and was negatively correlated with progression to type 1 diabetes (correlation = −0.39, p < 0.001). The final module (gray) consisted of 15 genes that were uncategorizable. The module–trait correlation heatmap is presented in **Figure 1D**. Heatmaps demonstrating the top DE gene expression levels for progressors versus non-progressors in each module are presented in **Supplementary Figures 3A–D**.

### Differentially expressed genes are enriched for diabetes-associated pathways

**Figure 2:**
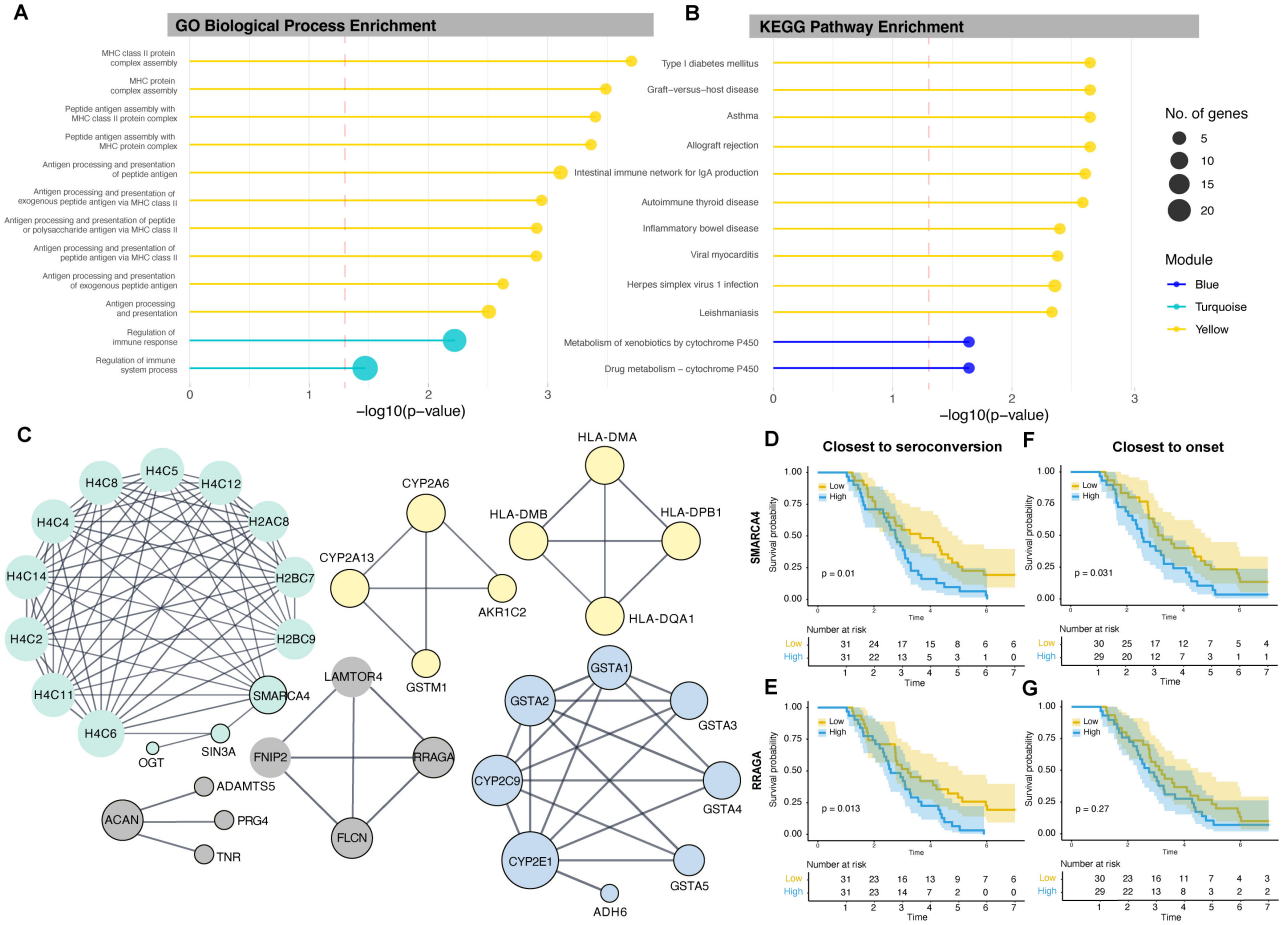
Lollipop plots depicting **(A)** significantly enriched GO biological processes for the yellow and turquoise modules and **(B)** significantly enriched KEGG pathways for the yellow and blue modules. The length of each lollipop corresponds to the significance level (−log_10_*p*), and colors denote the module. The dashed red line represents the significance threshold (p = 0.05; −log_10_ p ≈ 1.3). **(C)** PPI network linking proteins associated with DE genes. Nodes represent proteins, and edges represent known interactions. The size of each node indicates the degree of connectivity of the corresponding protein within the network, with larger nodes representing hub proteins that interact with more proteins. Nodes with black borders represent proteins from the input list. **(D, E)** Kaplan–Meier survival curves illustrating the rate of disease onset based on the expression levels of hub genes *SMARCA4* and *RRAGA* close to seroconversion, and **(F, G)** close to disease onset. The yellow line represents low gene expression and the blue line represents high gene expression, as determined by median expression values. GO, Gene ontology, KEGG, Kyoto Encyclopedia of Genes and Genomes.

To understand the biological and functional roles of each module, we calculated the enrichment of genes associated with gene ontology (GO) terms within each module using gene set enrichment analysis, focusing on biological processes. DE genes from the yellow module were largely enriched for terms related to antigen presentation by MHC class II. The turquoise module was enriched for the regulation of immune response and immune system processes (**Figure 2A**). The blue module did not yield any significant enrichment.

We repeated the analysis using KEGG pathways: the yellow module was significantly enriched for type 1 diabetes–related pathways as well as other immune and viral response pathways, while the blue module was enriched for drug metabolism and metabolism of xenobiotics by cytochrome P450 (**Figure 2B**). The turquoise module did not yield any significant enrichment.

### Identification of key proteins using protein-protein interaction networks

To better characterize the relationships between DE genes and their products, we constructed PPI networks to identify central node (hub) proteins with the highest degree in each module network that may be associated with disease development or progression (**Figure 2C**). The degree of a node is the number of edges directly connected to it, and nodes with high degree are considered hub proteins. In the yellow module, four hub HLA class II proteins—HLA-DMA, HLA-DMB, HLA-DQA1, and HLA-DPB1—formed a single network. The cytochrome P450 enzymes CYP2A6 and CYP2A13 were part of another network with the bile acid–binding protein AKR1C2 and the detoxification enzyme GSTM1. In the turquoise module, the chromatin remodeling protein SMARCA4 was a hub protein, and in the blue module, the cytochrome P450 protein CYP2E1 was identified as the hub. SMARCA4 had the highest degree of all the identified hub proteins, with multiple histones and the transcriptional corepressor SIN3A, which was linked with the enzyme O-linked N-acetylglucosamine transferase. The networks of each module were disconnected, suggesting different functions and independent regulatory mechanisms.

### *SMARCA4* and *RRAGA* expression stratifies type 1 diabetes progression post-seroconversion

We stratified progression to type 1 diabetes using Kaplan–Meier survival curves based on low or high gene expression (defined by median expression) of each hub gene. Participants with high expression of *SMARCA4* at the time closest to, but after, the age of seroconversion had a significantly increased rate of progression to type 1 diabetes (p = 0.010). Participants with high expression of *SMARCA4* measured closest to, but prior to, onset (p = 0.031) also showed higher progression, suggesting predictive value over time. Indeed, all non-progressors had low *SMARCA4* expression (**Figures 2D, E**) closest to, but after, the age of seroconversion.

Similarly, participants with high expression of *RRAGA* at observations closest to, but after, the age of seroconversion—but not prior to onset—had a significantly higher rate of progression (p = 0.013) (**Figures 2F, G**).

To assess *SMARCA4* and *RRAGA* as continuous variables, we performed Cox proportional hazards regression. Higher *SMARCA4* expression was significantly associated with increased risk of progression to type 1 diabetes (hazard ratio [HR] = 12.46, 95% confidence interval [CI]: 2.44–63.54, p = 0.002). However, when modeled as a continuous variable, *RRAGA* expression was not significantly associated with risk of progression to type 1 diabetes (HR = 2.11, 95% CI: 0.61–7.26, p = 0.236).

### Genes in protein-protein interaction networks connect to type 1 diabetes drug-gene targets

**Figure 3:**
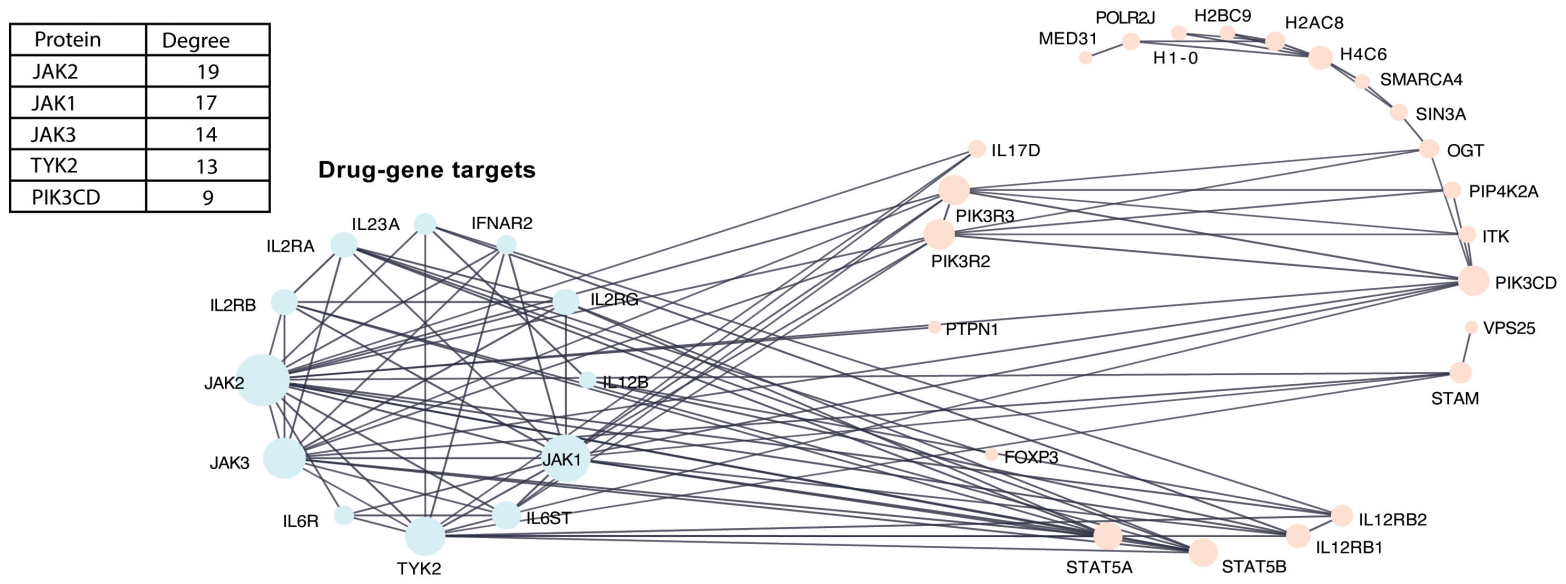
PPI network between DE genes with known type 1 diabetes drug-gene targets. PPI network integrating known drug–target interactions with DE genes to identify type 1 diabetes drug–gene targets. Nodes represent proteins, and edges represent known interactions. The accompanying table indicates the degree values of the top five proteins in the network.

PPI networks constructed between DE genes and previously identified type 1 diabetes drug–gene targets (*IL2RA, IL2RB, IL6ST, IL6R, TYK2, IFNAR2, IL12B, IL23A, IL2RG, JAK1, JAK2*, and *JAK3*) (13)—which have been targeted in clinical trials for autoimmune-mediated diseases—demonstrated extensive connectivity among these genes (**Figure 3**). All 12 genes in the drug target network connected with proteins and their interacting partners, which were primarily enriched in the turquoise module for immune signaling. Among the known drug targets, *JAK2* had the highest degree (interactions = 19).

### Immune cell populations are changing over time

**Figure 4:**
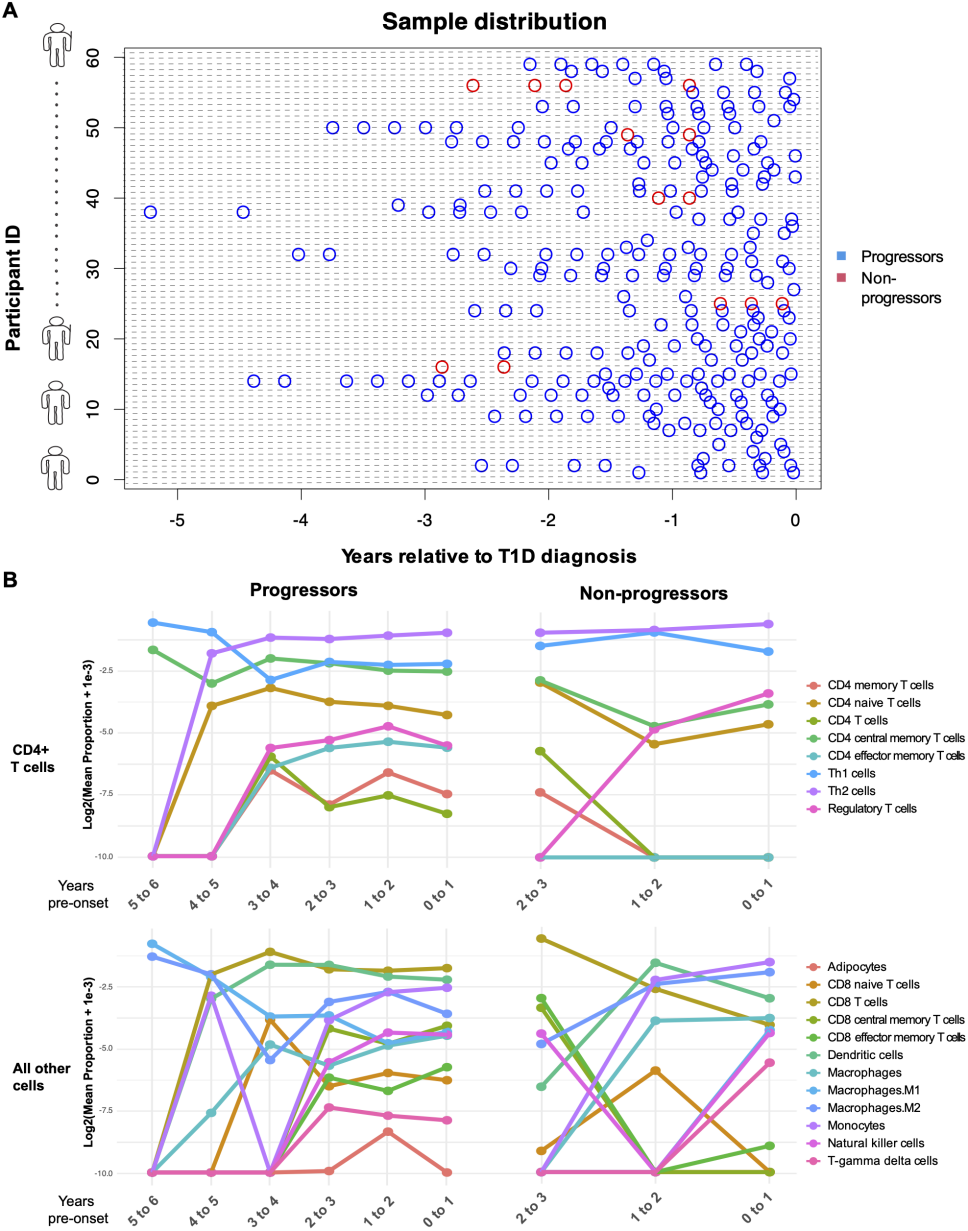
Computational enrichment of genes in progressors and non-progressors over time. **(A)** Plot of PBMC microarray data points used in the analysis. The blue points represent children who progressed to diabetes within the follow-up period of the study, and the red points represent non-progressors. The x-axis represents the time of sampling (years) relative to diagnosis of type 1 diabetes. Each subject on the y-axis is represented by a unique subject ID. **(B)** Line plots show the log_2_ -transformed proportions of immune cell types across binned age categories. The x-axis represents time (years) relative to diagnosis of type 1 diabetes. Post-onset in non-progressors is measured as the time of sampling subtracted by the mean age at diagnosis of those who progressed to diabetes.

To investigate the cellular context of transcriptomic changes, we applied a deconvolution approach to infer cellular composition and estimate the proportions of immune cell types from PBMC gene expression profiles by comparing sample transcriptional data with reference cell type–specific transcriptomes, enabling digital cytometry–based estimation of immune cell enrichment. The plot of PBMC microarray data points used in our analysis is shown in **Figure 4A**, and cellular deconvolution results are presented as line plots of log-transformed cell-type proportions across binned age categories relative to type 1 diabetes diagnosis in **Figure 4B**.

To determine whether immune cell types were significantly enriched or depleted between progressors and non-progressors, linear mixed-effects modeling was applied to the enrichment scores. CD4^+^ central memory T cells (estimate = 0.0147, p = 0.028) and CD4^+^ naïve T cells (estimate = 0.0070, p = 0.043) were significantly increased in progressors compared with non-progressors before false discovery rate (FDR) correction. However, this difference was not significant after correction.

### Gene signature distinguishes fast and slow progressors to type 1 diabetes

We used machine-learning models to identify a gene signature from DE genes that best distinguishes fast and slow progressors to type 1 diabetes. Of 56 progressors, 38 who progressed within ≤ 2 years after seroconversion were classified as fast progressors, while 18 who progressed > 2 years after seroconversion were classified as slow progressors. Linear mixed modeling with spline identified DE genes (n = 1,967) associated with fast or slow progression, while machine-learning models were used to derive gene signatures from these DE genes.

**Figure 5:**
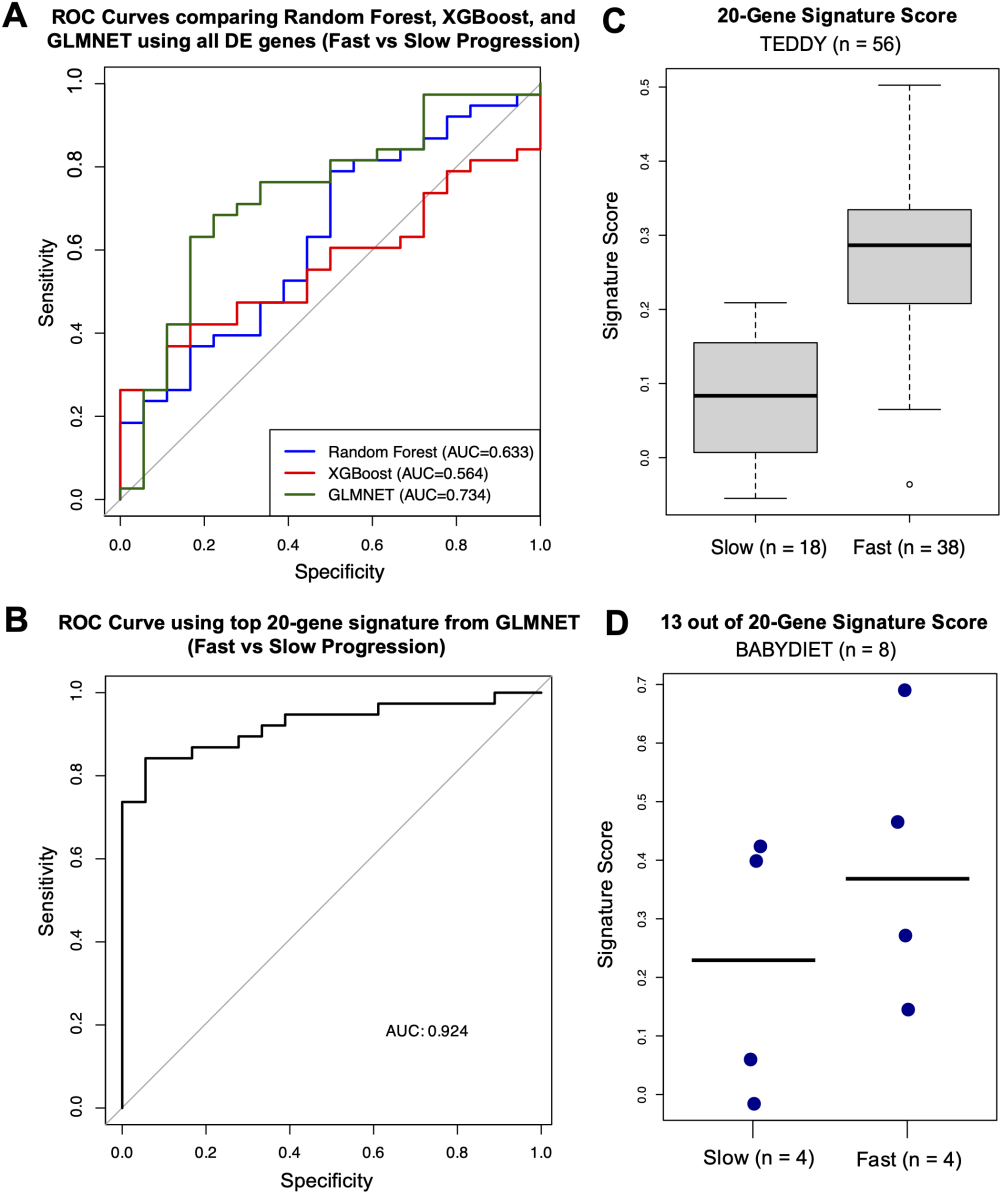
Machine learning analysis identifying gene signatures related to rate of disease progression. **(A)** ROC curves for machine-learning models (Random Forest in blue, XGBoost in red, and GLMNET in green) with corresponding AUC values. **(B)** ROC curve for the GLMNET-derived signature score distinguishing fast versus slow progressors with corresponding AUC value. **(C)** Boxplot of GLMNET-derived signature scores from the top 20 genes in fast and slow progressors. **(D)** Dot plot of signature scores in an independent validation cohort (BABYDIET) using 13 available signature genes, with a cut-off of 4.72 years post-seroconversion to categorize fast (n = 4) or slow progression (n = 4).

Using observations closest to—but after—the age of seroconversion, GLMNET demonstrated the highest discriminative performance in distinguishing fast from slow progressors (AUC = 0.734), outperforming both the Random Forest (AUC = 0.633) and XGBoost (AUC = 0.564) models when evaluated by leave-one-out cross-validation (**Figure 5A**).

Using the top 20 genes—*CACNA2D4, TCF7, USP36, DDI1, GSTT1, RUFY3, LCN8, C1ORF115, SMPDL3B, NCKAP1, SEMA6B, FAM118A, OR51E1, DOC2B, TREML4, ID1, LRRC37A4*, *MPND*, and *LYPD1*—identified by the GLMNET model (excluding poorly characterized genes), we established a robust classifier of progression rate (AUC = 0.924) (**Figure 5B**). The top 20 genes were selected to ensure an adequate number of features for validation in an external cohort with a limited gene set. A signature score, calculated as the difference between the mean expression of genes upregulated in fast progressors and the mean expression of genes upregulated in slow progressors, achieved strong discriminative performance. Higher scores indicate a gene-expression profile more similar to fast progressors, whereas lower scores reflect a profile more similar to slow progressors. Median signature scores of fast progressors were higher than those of slow progressors (**Figure 5C**).

### External validation using the BABYDIET confirms cohort confirms the fast versus slow gene signature

To externally validate our gene signature, we used PBMC gene-expression data from an independent cohort—the BABYDIET study—which included 18 autoantibody-positive children, 8 of whom progressed to type 1 diabetes. As the time to disease onset after seroconversion was longer in the BABYDIET cohort (median: 4.72 years, IQR: 3.27–5.22) than in the TEDDY cohort (median: 1.70 years, IQR: 0.85–2.40), we used a cut-off of 4.72 years to categorize fast (n = 4) or slow progression (n = 4) among the 8 progressors.

Due to limited gene availability in the cohort (N = 13,391), only 13 of the original 20 signature genes were present in the dataset (*CACNA2D4, TCF7, DDI1, GSTT1, RUFY3, LCN8, C1ORF115, SEMA6B, FAM118A, OR51E1, DOC2B, ID1*, and *LYPD1*). Nevertheless, even with fewer available signature genes, median signature scores of fast progressors remained higher than those of slow progressors (**Figure 5D**).

## Discussion

In this study, we evaluated transcriptomic contributors to progression from seroconversion to type 1 diabetes and identified DE genes and pathways predicting progression. After seroconversion, we found that the peripheral blood gene-expression profiles of progressors were distinct from those of non-progressors. The DE genes clustered into modules that correlated with various phenotypic traits associated with type 1 diabetes and were enriched for MHC class II and antigen presentation–related functions and regulation of immune response.

The yellow module, associated with the formation of MHC class II, the processing and binding of antigenic peptides to the assembled complex, and the presentation of these peptides via the MHC class II pathway to CD4^+^ T cells, was negatively correlated with the age at onset of type 1 diabetes and with disease development. Given that the autoimmune potential of T cells is strongly influenced by HLA class II–mediated editing of T cell receptor specificities during thymic selection, disruptions in these processes may contribute to disease development (14–17). Specifically, defects in MHC class II function could impair the elimination of autoreactive T cells in the thymus, allowing them to escape into the peripheral immune system, where they may recognize pancreatic β-cell antigens and initiate autoimmune destruction. The enrichment of the yellow module in type 1 diabetes–related pathways further supports the potential role of DE genes in disease regulation.

The turquoise module, broadly associated with the regulation of immune response and immune system processes, encompassing both immune activation and suppression, was positively correlated with progression to type 1 diabetes. In the context of type 1 diabetes, this correlation likely reflects ongoing immune responses and concomitant immune regulation, resulting in chronic islet inflammation and dysregulation of central and peripheral immune tolerance (18). On the contrary, the blue module, which did not show any significant gene ontology enrichment, was negatively correlated with progression to type 1 diabetes. Consistent with this, the blue module includes known type 1 diabetes–associated genes such as *FOXP3* and *FOXO3*. The blue module was enriched in KEGG pathways related to drug metabolism and the metabolism of xenobiotics via cytochrome P450, consistent with previous studies linking cytochrome P450 activity to environmental exposures such as polycyclic aromatic hydrocarbons (19), heavy metals (20), and pesticides (21), as well as to β-cell dysfunction and death (22). Interestingly, cytochrome P450 is also associated with the metabolism of many drugs, and its inhibition or induction can result in significant drug–drug interactions (23). This suggests a potential link between environmental exposures and metabolic processing in disease pathogenesis.

Using PPI networks built upon DE genes in each module, several hub proteins—HLA-DMA, HLA-DMB, HLA-DPB1, HLA-DQA1, CYP2A6, CYP2A13, CYP2E1, and SMARCA4—were identified. HLA-DM catalyzes the release of CLIP, a placeholder peptide, from the MHC II binding groove and edits MHC II-bound peptides to allow presentation of peptide antigens by MHC II to T cells (24, 25). In adults with type 1 diabetes, cell-surface MHC II–CLIP was increased compared with controls, suggesting fewer HLA-DM molecules and fewer MHC II–peptide complexes formed. Furthermore, in individuals with residual C-peptide, HLA-DMA and HLA-DMB were increased compared with those without, suggesting changes in antigen presentation over time (26). Type 1 diabetes risk allomorphs HLA-DQ2 and HLA-DQ8 were shown to be less susceptible to DM activity (27, 28). Together, these data suggest an important role for MHC II–peptide-associated peripheral tolerance in progression to diabetes post-seroconversion in genetically high-risk children.

The genes C*YP2A6, CYP2A13*, and *CYP2E1* belong to the cytochrome P450 gene family, which encodes enzymes involved in the metabolism of drugs, toxins, and endogenous compounds (29). Analysis of the PPI network suggests involvement in bile acid metabolism. The *SMARCA4* gene, through the BRG1 protein, is part of the SWI/SNF chromatin-remodeling complex, which regulates gene expression and has been shown to cause pancreatic hypoplasia, glucose intolerance, hyperglycemia, and impaired insulin secretion in mice when specifically knocked out in β cells (30). *SMARCA4* was linked to multiple histones, the corepressor SIN3A, and the enzyme O-linked N-acetylglucosamine transferase, which has multiple regulatory roles, including regulation of insulin sensitivity (31).

A more in-depth analysis of hub genes revealed that the expression of *SMARCA4*, a DE gene, and *RRAGA*, a protein interactor included during PPI-network construction, stratified progression to type 1 diabetes after seroconversion. However, only *SMARCA4*, and not *RRAGA*, stratified progression at observations close to, but prior to, onset. This suggests that *SMARCA4* is involved in both early and late stages of progression and likely has a sustained role in immune modulation, whereas *RRAGA* may play an early role in disease progression. However, the biological significance of *RRAGA* remains to be determined given its lack of differential expression between progressors and non-progressors.

DE genes in the PPI networks were also highly connected to type 1 diabetes drug–gene targets (15), particularly JAK2 and JAK1. Dysfunction in the JAK–STAT signaling pathway has been implicated in the pathogenesis of various metabolic diseases, including metabolic syndrome and diabetes (32), and the JAK1/2 inhibitor baricitinib was shown to preserve β-cell function in a phase 2 clinical trial for stage 3 diabetes (32). However, we acknowledge that protein interactions may be limited by PPI databases, and the observed differences could reflect missing interactions rather than true biological variation.

While cellular deconvolution did not reveal any DE immune populations between progressors and non-progressors after FDR correction—likely due to class imbalance—CD4^+^ T central memory cells and CD4^+^ naïve T cells demonstrated a nominal increase in progressors, which warrants further investigation in larger cohorts.

This study has several limitations. The small number of non-progressors after filtering for non-sero-revertors represents a key limitation, as this imbalance may reduce statistical power to detect differences and increase the potential for model bias or overfitting. However, because small samples inherently have less power to detect true effects, false negatives are more likely than false positives. Due to this class imbalance, we restricted our machine-learning methods to identifying gene signatures predictive of the rate of progression to type 1 diabetes. Although the 20-gene signature demonstrated strong discriminative power, validation using the BABYDIET cohort was limited by both cohort size and gene availability, restricting the statistical power to accurately assess signature-score differences between progression groups. Despite this, slow progressors in the BABYDIET cohort had lower median signature scores, presenting a preliminary, supportive trend of the gene signature. Given these limitations and our sensitivity analysis, confirmation in a larger, independent cohort with more complete gene coverage to robustly validate the gene signature is an essential next step.

In summary, our analysis revealed that autoantibody-positive children who progressed to type 1 diabetes displayed distinct peripheral blood gene-expression profiles compared with non-progressors, forming co-expression gene modules associated with MHC class II–related functions and immune-regulation pathways. We also found that the rate of progression among progressors can be stratified using a gene expression–based signature, highlighting transcriptional differences between fast and slow progressors.

## Data Availability

The original contributions presented in the study are included in the article/**Supplementary Material**. Further inquiries can be directed to the corresponding author.
