# Supplementary material for: Peripheral blood gene expression stratifies rate of progression to type 1 diabetes in autoantibody-positive children in the TEDDY study
Source: Front Immunol. 2025 Nov 11;16:1703839. doi: 10.3389/fimmu.2025.1703839 (PMC12645633; doi:10.3389/fimmu.2025.1703839)
Supplement: Supplementary file 1 [file Table1.docx]

**Supplementary Figure 1. Schematic illustration of the TEDDY cohort.**

(A) Inclusion and exclusion criteria used to select the subset of 62 participants that were autoantibody positive. (B) Observations demonstrated prospectively for the 62 participants, with each line representing the longitudinal measurements from a single participant. Blue dots indicate progressors to type 1 diabetes, while red dots indicate non-progressors. Orange dots indicate the age at islet autoantibody seroconversion and pink dots mark the age at T1D onset for progressors.

**Supplementary Figure 2. Gene dendrogram and module assignment with subclustering of yellow module**

Gene dendrogram generated using average linkage hierarchical clustering. The coloured row beneath the dendrogram indicates module assignments identified by the Dynamic Tree Cut algorithm in WCGNA. Four modules, yellow (split into 2 major subclusters), turquoise, blue, and grey were constructed from differentially expressed genes.

**Supplementary Figure 3. Heatmaps demonstrating differentially expressed gene expression levels for group effect measured relative to type 1 diabetes diagnosis in each module.**

(A) Top 100 most significant genes in progressors versus. non-progressors in the yellow module. (B) Top 100 most significant genes in progressors versus. non-progressors in the turquoise module. (C) Top 100 most significant genes in progressors versus. non-progressors in the blue module. (D) Most significant genes in progressors versus. non-progressors in the grey module. Dotted line separates progressors from non-progressors.
